# Alternative Bedding Materials for Poultry: Availability, Efficacy, and Major Constraints

**DOI:** 10.3389/fvets.2021.669504

**Published:** 2021-08-17

**Authors:** Siaka Diarra, Sonny Lameta, Falaniko Amosa, Sanjay Anand

**Affiliations:** School of Agriculture, Geography, Environment, Ocean and Natural Sciences, The University of the South Pacific, Apia, Samoa

**Keywords:** bedding material, bird welfare, housing systems, product quality, poultry production

## Abstract

The increasing demand of wood shavings (WS) and sawdust (SD) by other industries and growing concerns of potential chemical contaminants from wood products have amplified research interest in alternative bedding materials for commercial poultry. Several alternative materials—corn cob (CC), straws (ST) and hays (HA), sand (SA), shredded papers (SP), rice hulls (RH), peanut hulls (PH), and gypsum (CaSO_4._2H_2_O_2_)—can replace conventional ones in poultry houses, depending on availability, cost, and ability to absorb and adsorb moisture and provide the birds enough room to exhibit their natural behaviors. Alternative materials hold a brighter future as bedding materials, but more studies about their physicochemical properties and litter management practices for optimum poultry welfare are recommended.

## Introduction

Globally, the poultry industry is witnessing a rapid growth to meet the demand of the ever-increasing world population with higher income and better food choices (1, 2). While commercial layers may still be found in cages, broilers are almost always raised on litter. The rapid growth rate in broiler production (1) and the gradual ban of the cage system for layers will mean more litter materials for the poultry industry. Several factors including unavailability, increasing cost, and possible health and safety risks of conventional materials have been the major forces driving research into new bedding materials for commercial poultry. Wood shavings (WS) and sawdust (SD) are becoming scarce and expensive (3–5) due to their increasing use for highway construction (6–8), lightweight concretes (9–12), and heating and roofing felts (5, 13).

The past decades have seen increased research in alternative litter materials for poultry (14). Several alternatives to wood by-products have been used with varying outcomes on bird welfare and performance. In view of differences in the availability of substrates used for bedding materials among regions, reviews summarizing the characteristics of alternative materials, their effectiveness, and major issues would benefit the poultry industry. This paper reviews the availability, efficacy, and major issues in the use of selected alternative bedding materials in commercial poultry houses.

## Some Attributes of Litter Materials

For a product to qualify as litter material, it must be readily available, cheap, absorbent, and free from dust and contaminants; must have low thermal conductivity; and should not cake or compact. The type of bedding material exerts direct effects on productive performance (15, 16), health (17, 18), product quality (carcass and eggs), and poultry welfare (14, 15, 19, 20).

A good litter material should be able to absorb and release moisture to the environment as quickly as possible (4). Several chemical characteristics including the cellulose, silica, and lignin contents influence the quality of litter materials. Cellulose and silica are capable of absorbing water due to their high hydrophilic groups and higher surface area due to smaller particle size (21–23), respectively. The initial moisture content is also an important factor to consider in the choice of any bedding material. High moisture in the bedding increases ammonia build-up through increased microbial metabolism, resulting in respiratory and eye lesions (20, 24), which adversely affect birds' welfare and productivity (25). Wet litter predisposes to breast blisters and hock burns (25, 26), which reduce carcass quality in meat birds. Litter moisture (27–29) and source may also encourage the multiplication of *Salmonella, Campylobacter*, and *Listeria* spp. and *Eimeria* spp., the causative agent of coccidiosis (18). In addition to bird health and product quality, nitrogen (N) loss through ammonia volatilization is a major air pollutant with severe environmental health consequences (30).

Because poultry may consume a significant proportion (as much as 4%) of their feed in litter (15, 17) or forage directly on the litter material (1, 31, 32), the bedding substrate must be free from possible contaminants that could be taken up in the tissues. Chemical preservatives have been major criticisms to the use of WS and SD as bedding materials. Common wood preservatives such as sodium borate and copper chrome arsenate (C.C.A.), may be retained in the tissue, posing health risks to the human consumer of poultry products (1). As a measure to minimize this risk, many countries in Europe (France, Ireland, England, and Denmark) are now producing untreated softwood shavings as bedding material for the intensive livestock and poultry industries (1). Using suitable litter materials, stocking density and litter management practices have the greatest influence on poultry behavior, welfare, and productivity (33, 34). The quality of the litter is determined by the litter moisture, pH, ammonium nitrate content, caking level, and water holding capacity (35).

Dustiness from extremely dry bedding materials (17) or very fine particles (15) may predispose birds to respiratory problems, resulting in higher mortality. Very large and coarse bedding materials may, however, downgrade carcass quality due to their abrasive effects (20, 36). From the foregoing discussion, several characteristics of the bedding material affect poultry welfare, productivity, and product quality.

## Availability of Selected Alternative Bedding Materials For Poultry

### Rice Hulls

Rice hull (RH) is an important by-product of the rice milling process, representing about 25% of rice paddy (37, 38). This will translate to about 179 million tons of hulls from the estimated 715 million tons of paddy produced globally per year (38). RH is still a waste in many regions, posing disposal problems, and this has increased research interest in alternative uses. RH is burnt onsite to provide energy for mechanical rice milling, used in the ceramic and construction industries (39, 40) or livestock and poultry feeding, but its high silica and lignin contents (41) limit its full utilization as feed, making it available as bedding material in many rice-growing regions.

### Corn Cob

Corn or maize (*Zea mays*) is the most important cereal with the global production estimated at about 875 million tons (42). Corn cob (CC), a by-product of corn processing for grain, accounts for 180–200 kg per ton of grains produced (43). Currently, this residue has a number of limited applications including building material and activated carbon (44, 45) and still regarded as waste in many producing regions, posing environmental problems (46). CC has high holo-cellulose (cellulose and hemicellulose) and low lignin contents (47). The ready availability and high absorbency (4, 26) make CC a potential litter material for poultry.

### Sand

The annual global consumption of sand (SA) is estimated to be 15 billion tons (48). Sand is increasingly used by several industries, including the building industry (houses and roads), electronics (computer chips and microprocessors), cosmetics, and detergents, among others. River sand is clean and has high water absorbing capacity but desert sand is too fine and smooth, high in clay, iron oxides, and lime but lacks silicon dioxide (4, 49, 50), making the former a better litter material than the latter. The poor absorbency of desert sand and the increasing demand of river sand for bedding and the construction industry may, however, exacerbate the already existing environmental consequences of sand mining (48, 51).

### Straws/Hays

Straw (ST) is the fibrous residue from grain crop harvest. As the global production of cereals such as rice, wheat, and barley continues to increase, ST from the harvest of these crops become readily available. ST are, however, high in lignin (52) and low in hydrophilic groups (53), which reduce their ability to absorb and release moisture. Hay (HA) from grasses, although lower in lignin compared to ST, is also not very absorbent probably due the relatively high initial moisture content in cured HA (10–15%). Straw and HA beddings may therefore compact rapidly, predisposing birds to fungal infection and increasing the incidence of breast blisters. Where ST and HA are cheap, however, they may be used as bedding materials to save cost, provided the litter is renewed regularly. The ease to compost ST could be an environmental incentive as this will reduce disposal problems of straw-based bedding materials.

### Sugar Cane Bagasse

Sugar cane bagasse (SCB), a by-product of sugar refining, amounts to about 140 kg per ton of sugar cane processed (54). SCB has high water absorbing ability due to its residual sugar content. However, SCB cakes easily (55), and this calls for more research to improve its utilization as bedding material for poultry.

### Shredded Newspapers

Newspapers have long been used as animal bedding. Despite the rapid adoption of soft copies, papers are available (3) and may pose disposal problems (56). Paper is absorbent, is easy to decompose (57), and has minimal health risks (free from dust, contaminants, and pathogenic organisms), but does not release moisture to the environment as quickly as possible. Processing newspapers into chips improves its moisture holding capacity and evaporative loss (58). However, the growing trend of the paper recycling industry may not spare this for use as economic bedding material. Paper recycling increased from 5 million tons in 1960 to 44 million tons in 2017 (48). Consumption of newspapers printed with inks based on petroleum-laden or heavy metals (59, 60) may, however, pose health concerns.

### Peanut Hulls

Globally, peanut (*Arachis hypogaea*) occupies, on average, 22.2 million hectares, with a share of 16.3 million hectares, 7.39 million hectares, and 0.7 million hectares in Asia, Africa, and South and Central America, respectively (61). Peanut yield ranges from 3 to 4 ton/ha, but yields as high as 9.6 tons/ha have been reported (62). Global peanut production was estimated at 40 million tons in 2015 (63). With the estimated 25% hull (64, 65), this will amount to about 10 million tons of peanut hull (PH) consisting of variable amount of broken kernels. In most peanut-producing countries, the hulls are burned on farm, dumped, or allowed to deteriorate naturally (66), making this by-product readily available for various uses.

### Gypsum

Gypsum (CaSO_4._2H_2_O_2_) is a carbonaceous material used in the wallboard and cement industries as well as for agricultural production. However, global gypsum production is reported to exceed the capacity of these industries (67). This has increased research into alternative uses for gypsum to minimize problems of storage and disposal. Because of its ability to absorb moisture and reduce litter pH, the application of gypsum in litter amendment has been receiving research attention.

## Summary of The Application of Some Alternative Materials

### Bedding Material and Litter Quality

There is sufficient literature on the effect of bedding substrate on litter quality. Benabdeljelil and Ayachi (16) evaluated whole and ground wheat straw (WHS), ground rice straw (RS), RH, SD, and WS singly or in combinations in Warren cockerel chicks and found no effect on water consumption, but litter moisture, temperature, pH, and overall quality score were reduced on straw-based litters compared to the other materials. The authors attributed this to the low water holding capacity (WHC) of the straw-based litters due to the high lignification of straw. The insolubility of lignin is mainly due to its compact nature, low molecular weight, and fewer hydrophilic groups (53). In another report, Diarra et al. (68) found no differences between WS and whole or chopped Para grass (*Brachiaria mutica*) hay in terms of moisture retention and litter caking in Shaver brown laying hens. Grimes et al. (69) also observed that chopping improved the efficacy of straw as a bedding material and concluded that particle size rather than type of material is an important factor of litter quality. The effect of particle size of fibrous materials on WHC is well-documented (52, 70, 71). Grasses for hay production are normally harvested at relatively younger age with lower lignin content compared to straw. Low lignification and high cellulose content may explain the higher WHC of hay. It follows, therefore, that grasses used for bedding should be harvested when the ratio cellulose/lignin is still high. However, as cured hay may contain up to 20% moisture (72, 73), this initial moisture content will affect its ability to absorb moisture for a longer period, but needs more investigation. In a study comparing different bedding materials (WS, SA, RH, grass, SP, and CC) for broilers, Garcês et al. (4) found no difference in water-releasing capacity among the materials during the first 24 h, but the ability of WS, RH, grass, and SP to lose water reduced by about 34% thereafter. This suggests that the duration of rearing and birds' age are important considerations in assessing the quality of a bedding material for poultry. CC and SA litters had the lowest moisture content at the conclusion of the experiment. The ability of CC to absorb and lose water has been attributed to its higher content of cellulose and hemicellulose and lower lignin (74). The high water absorption capacity of sand has been attributed to its coarse particle sizes, which release water faster and keep the surface dry (4). Contrary to these findings, however, Shields et al. (26) observed no differences in litter moisture content and temperature between SA and WS. Gypsum is reported to reduce litter NH_3_ content (75–77), which could improve the welfare of birds. Several factors including litter depth, relative humidity, and bird stocking density might be possible reasons of variation among studies. The relative humidity greatly affects the ability of a bedding material to lose water to the environment. Weaver and Meijerhof (78) observed increased litter caking, moisture, and ammonia contents with increasing relative humidity from 45 to 75% in environmentally controlled broiler houses.

### Bedding Materials and Poultry Welfare and Performance

The quality of bedding materials has the greatest influence on ammonia production, which adversely affects the performance of birds. Studies have shown that high levels of ammonia in the house decrease the efficiency of feed utilization, weight gain, and egg production (79–83). The mode of action of ammonia on poultry performance is directly related to its damaging effect on the respiratory tissues (84) and impairment of the bird's immune response (85). Microbial activity on wet litters (86–89) is a major factor in ammonia production in the house. It is therefore evident that the quality of the bedding material, mainly its ability to absorb moisture, is important in maintaining a healthy house environment and better performance of poultry. As different bedding substrates produce different conditions in the poultry house (20, 26, 90), choosing the right litter materials is important for maximum poultry welfare and productivity. Because litter moisture is an important factor encouraging the multiplication of pathogenic organisms (27–29, 88), it follows that bedding materials with low WHC would quickly predispose to disease outbreak.

Benabdeljelil and Ayachi (16) observed no effects of whole and ground WHS, ground rice straw (RS), RH, SD, and WS or their combinations on growth, water consumption, and mortality rate in Warren cockerel chicks, but litter ammonia content was increased on straw, probably due to its low WHC earlier mentioned. Monira et al. (91) observed improved body weight gain (BWG), feed consumption, and survivability in broilers raised on SD compared to birds on RH, SCB, and WHS beddings. Contrary to these findings, Toghyani et al. (92) found no effects of WS, RH, paper roll (PR), and sand on feed intake, feed conversion, and mortality of broilers, but BWG and antibody titer against Newcastle disease reduced on RH. The authors attributed this to possible higher pathogenic bacterial count on RH compared to the other materials. Munir et al. (93) also confirmed lower count of enteric bacteria on sand compared to organic materials due mainly to differences in nutrient availability and lack of binding site for bacteria in sand.

Nowaczewski et al. (94) reported better BWG, foot health, feed conversion ratio, hemoglobin saturation, and lower mortality rates on WS compared to whole or chopped WHS beddings. Several studies (4, 26, 74) found no difference in ammonia content between WS, CC, and SA litters in broilers. Sand bedding may also improve bird welfare through increased behavioral performance (26, 74). Given the choice, broilers showed preference for SA to WS (20, 26, 74) or RH, chopped Napier grass, and SCB (20) in terms of performance and natural behaviors. Diarra et al. (68) also found no differences between WS and whole or chopped Para grass hay on egg performance and litter caking in laying hens, but chopping the hay reduced the incidence of feather pecking, probably due to the inability of the birds to forage efficiently on larger particle sizes. Several factors including the class and age of the bird, stocking density, litter processing, and thickness all affect the suitability of a material as bedding substrate. Higher stocking densities reduce litter quality and bird welfare (25, 95) and bird performance (96, 97) due to higher excreta output and rapid deterioration of the bedding material. Shao et al. (98) also observed an improved welfare and production of broiler chickens with increasing thickness of SD-based beddings from 4 to 16 cm. These findings suggest that increasing stocking density must be accompanied by corresponding increases in litter depth.

Ramadan et al. (5) found no effects of WS, whole RS, and SA singly or in combinations on carcass weight and welfare indices (fear, developmental instability, feather score, footpad dermatitis, and stress) of broilers, but BWG improved on SA-based litters. Gizzard weight increased on WS compared to SA or RS beddings. The improved weight gains on SA or mixtures containing it may be attributed to better welfare, further elucidating the quality of SA as bedding material. The authors attributed the pattern of gizzard development to (i) increased activity of this organ due to consumption of the fibrous shavings, (ii) faster rate of passage of SA through the gastrointestinal tract, and (iii) difficulty in consuming RS compared to WS.

The use of PH as litter material is well-documented (58, 99, 100). Lien et al. (58) found no differences in litter pH, BWG, feed consumption, mortality, and flock uniformity of broiler breeder pullets raised on PH and fine WS from 11 weeks of age, but gizzard weight increased on WS than PH. The authors attributed this to litter consumption and differences in fiber content between the litter sources. According to Jones et al. (99), aflatoxin contamination can be a major problem in birds reared on PH in early life, but the addition of moisture and fecal materials to the bedding with age or aflatoxin breakdown by ammonia will overcome this. These observations suggest the need for more research in stocking densities, which will produce maximum dropping on PH in early life. Using SA, WS, and SP as litter material had no marked effects on broiler performance but treatment with bentonite reduced litter moisture content (101) due to high ability of bentonite to absorb moisture. Where materials with low moisture absorbing capacity are readily available, litter treatment can be a viable option.

The mode of action of gypsum on NH_3_ production is not clear but probably by inactivation of NH_3_-producing bacteria through absorption of litter moisture content causing osmotic stress (102) and reduction in litter pH (103). In a 49-day growth experiment, Sampaio et al. (104) observed a significant reduction of bacterial count in broiler litter amended with gypsum. Oliveira et al. (103) reported NH_3_ reduction in broiler litter treated with 40% gypsum. In another study, addition of gypsum to broiler litter at 10 and 20% reduced its NH_3_ content by 21% (76). These results suggest that several factors may affect the efficacy of gypsum as litter material. Phosphorus emission from poultry litter, another environmental concern, is also reported to be minimized by gypsum application (67). The effect of gypsum-treated litter on live performance is not consistent. Grimes et al. (105) observed no effect of a mixture of gypsum, cotton waste, and old newspapers on the growth performance of broilers and turkeys. Wyatt and Godman (106) reported a significantly lower body weight in broilers raised on recycled wallboard gypsum compared to the control based on wood shavings. Several factors including the source of primary bedding material, litter depth, and application rate of gypsum may all affect the performance of poultry. This calls for the need to do more research in the application of gypsum as bedding material for poultry. Table 1 summarizes the efficacy of selected materials as bedding substrates in poultry houses and the major issues.

**Table 1:** Summary of the potential and constraints of selected substrates as litter materials for poultry.

**Material**	**Potential**	**Constraints**	**References**
Wood shavings	Conventional litter material, highly absorbent, and adsorbent. Improves welfare through dustbathing and foraging.	Expensive due to competition among several industries. Potential risks of contamination from chemically treated woods.	(1, 6)
Sawdust	Absorbent and improves welfare through dustbathing and foraging.	Competition with other industries, low ability to release moisture and frequent caking. Fine particles may predispose to respiratory problems. Risks of chemical preservatives.	(56, 107)
Straw	Readily available and cheap. Chopping improves water holding capacity and provides for foraging.	Low water holding capacity due to lignin content and risk of caking.	(52, 55, 69)
Sand	Readily available. Coastal sand absorbent and adsorbent. Clean bedding with no risk of caking. Maximizes bird welfare through dustbathing.	Environmental consequences of sand mining. Desert sand has poor absorbency.	(4, 26, 74)
Corn cob	Readily available during corn harvest. Absorbent and adsorbent. Reduces litter ammonia content and improves bird welfare and productivity.	Energy cost of crushing corn cob. Future competition due to increasing use as ruminant feed.	(4, 26)
Rice husk	Available and cheap. Reduces the incidence of footpad dermatitis and breast blisters.	May easily compact.	(4, 18, 101)
Shredded newspapers	Readily available and cheap from paper industries.	Low absorbency. Easily cakes and causes breast blisters.	(55, 56)
Sugar cane bagasse	Readily available from sugar industries. Absorbent and adsorbent.	May cake and cause breast blisters.	(55)
Corn silage	Reduces the incidence of salmonella.	Competition with the ruminant industry.	(108)
Coconut husk	Readily available and cheap.	Low water holding capacity	(4)
Peanut hull (shell)	Readily available and may pose environmental problems. Comparable to shavings as litter material.	Risk of aflatoxin contamination.	(58, 64, 100, 109)
Gypsum	Readily available, cheap and absorbent. Reduces bacterial load, NH_3_, and phosphorus emissions	May have no beneficial effects on growth.	(67, 76, 102, 106)

### Some Nutritional and Environmental Factors Affecting Litter Quality

Several nutritional and environmental factors may also influence litter quality and bird performance. Diet composition and physicochemical characteristics are known to influence litter quality in birds kept on the same bedding substrate (43, 110–113). Viscous fibers in the diet reduce nutrient digestion and absorption (111, 112, 114) and increase cecal fermentation (115, 116). Increased fermentation in the cecum resulting in soft feces is a major cause of wet litter in poultry houses (110). This suggests the need to increase litter depth to cope with excessive moisture in the excreta of birds fed high viscous fiber diets. Several salts including NaCl and KCl, which are added to the feed and water as a means of combatting heat stress, would also increase excreta output and litter moisture (117). High humidity in the air also reduces the ability of the bedding material to lose moisture to the environment and adversely affects poultry performance. Weaver and Meijerhof reported higher BWG and drier moisture in broiler chickens at 45% compared to those kept at 75% relative humidity.

## Conclusions

Wood-based materials, the traditional litter materials in many regions, are becoming short in supply or criticized due to growing concerns of potential chemical contaminants. Several materials including newspapers, CC, sand, RHs, peanut shell, and gypsum can be suitable alternatives to wood-based beddings in poultry houses. The source, texture, and particle size of the material; litter depth; age and class of bird; duration of rearing; diet composition; stocking density; and relative humidity all affect the suitability of a material as litter. There is a need for more research on litter management practices and cost-effectiveness of different materials for optimum bird welfare, productivity, and product quality.

## Author Contributions

SD: conception, investigation, methodology, writing—original draft and writing—review and editing. SL, FA, and SA: writing—review and editing. All authors contributed to the article and approved the submitted version.

## Conflict of Interest

The authors declare that the research was conducted in the absence of any commercial or financial relationships that could be construed as a potential conflict of interest.

## Publisher's Note

All claims expressed in this article are solely those of the authors and do not necessarily represent those of their affiliated organizations, or those of the publisher, the editors and the reviewers. Any product that may be evaluated in this article, or claim that may be made by its manufacturer, is not guaranteed or endorsed by the publisher.
